# Emergence of carbapenem-resistant *Klebsiella pneumoniae* harbouring *bla*_OXA-48_-like genes in China

**DOI:** 10.1099/jmm.0.001306

**Published:** 2021-01-28

**Authors:** Ying Chen, Li Fang, Yunxing Yang, Rushuang Yan, Ying Fu, Ping Shen, Dongdong Zhao, Yan Chen, Xiaoting Hua, Yan Jiang, Robert A. Moran, Willem van Schaik, Yunsong Yu

**Affiliations:** ^1^​ Department of Infectious Disease, Sir Run Run Shaw Hospital, Zhejiang University School of Medicine, Hangzhou, PR China; ^2^​ Key Laboratory of Microbial Technology and Bioinformatics of Zhejiang Province, Hangzhou, PR China; ^3^​ Regional Medical Center for National Institute of Respiratory Diseases, Sir Run Run Shaw Hospital, Zhejiang University School of Medicine, Hangzhou, PR China; ^4^​ Department of Clinical Laboratory, Affiliated Hangzhou First People’s Hospital, Zhejiang University School of Medicine, Hangzhou, PR China; ^5^​ Department of Clinical Laboratory, Sir Run Run Shaw Hospital, Zhejiang University School of Medicine, Hangzhou, PR China; ^6^​ State Key Laboratory for Diagnosis and Treatment of Infectious Diseases, Collaborative Innovation Center for Diagnosis and Treatment of Infectious Diseases, The First Affiliated Hospital, School of Medicine, Zhejiang University, Hangzhou, Zhejiang, PR China; ^7^​ Institute of Microbiology and Infection, University of Birmingham, Birmingham, UK

**Keywords:** OXA-181, OXA-232, *Klebsiella pneumoniae*, carbapenem-resistant *Klebsiella pneumoniae*, China

## Abstract

*
Klebsiella pneumoniae* strains carrying OXA-48-like carbapenemases are increasingly prevalent across the globe. There is thus an urgent need to better understand the mechanisms that underpin the dissemination of *bla*_OXA-48_-like carbapenemases. To this end, four ertapenem-resistant *K. pneumoniae* isolates producing OXA-48-like carbapenemases were isolated from two patients. Genome sequencing revealed that one sequence type (ST) 17 isolate carried *bla*_OXA-181_, whilst three isolates from a single patient, two ST76 and one ST15, carried *bla*_OXA-232_. The 50514 bp *bla*_OXA-181_-harbouring plasmid, pOXA-181_YML0508, was X3-type with a conjugation frequency to *Escherichia coli* of 1.94×10^−4^ transconjugants per donor. The *bla*_OXA-232_ gene was located on a 6141 bp ColKP3-type plasmid, pOXA-232_WSD, that was identical in the ST76 and ST15 *K. pneumoniae* isolates. This plasmid could be transferred from *K. pneumoniae* to *E. coli* at low frequency, 8.13×10^−6^ transconjugants per donor. Comparative analysis revealed that the X3 plasmid acquired the *bla*_OXA-48_-like gene via IS*3000*-mediated co-integration of the ColKP3-type plasmid. Our study highlights how plasmid integration and rearrangements can contribute to the spread of *bla*_OXA-48_-like genes, which provides important clues for clinical prevention of the dissemination of *K. pneumoniae* strains carrying *bla*_OXA-48_-like carbapenemases.

The emergence and prevalence of carbapenem-resistant *Klebsiella pneumoniae* is an increasingly significant threat to public health worldwide and the carbapenemases OXA-48 and its derivatives contribute to the spread of carbapenem resistance in this important opportunistic pathogen [1]. OXA-48 was first found in *K. pneumoniae* in Turkey [2], and its derivatives such as OXA-181 [3], OXA-232 [4] and OXA-163 [5] were subsequently found in European hospital outbreaks [6]. OXA-48-like carbapenemases appear to have disseminated rapidly via the actions of mobile genetic elements [7]. Broad host range L/M-type and ColE1-like plasmids commonly bear *bla*_OXA-48_-like genes in *Enterobacteriaceae* [8], and the *bla*_OXA-48_-like genes are associated with many types of insertion sequences and transposons. For example, the *bla*_OXA-181_ gene has been mobilized by IS*Ecp1* and the resulting structure has been called Tn*2013* [9]. However, the mechanisms of *bla*_OXA-48_-like gene dissemination and how these important enzymes are spreading remains unclear.

To explore these unclear mechanisms, four *K. pneumoniae* isolates were collected from rectal swabs or faecal samples taken for surveillance from two patients in Sir Run Run Shaw Hospital (Zhejiang, China) between May and December 2018. YML0508 was isolated from a 60-year-old female while WSD411, WSD2016 and WSD2080 were isolated from an 84-year-old male (Table 1). Antimicrobial susceptibility testing showed that all four isolates were resistant to ertapenem and WSD411 was extensively-drug resistant, only being sensitive to colistin from the panel of antibiotics used in susceptibility testing (Table 2) [10]. Sanger sequencing of PCR products amplified using published primers that detect *bla*_OXA-48_-like genes [11] revealed that YML0508 contained *bla*_OXA-181_, while the remaining isolates contained *bla*_OXA-232_. Strains YML0508, WSD2016 and WSD2080 showed a similar carbapenem susceptibility spectrum, only resistant to ertapenem but sensitive to imipenem and meropenem. As many OXA-48-like producers exhibited only decreased susceptibility to one of the carbapenems, they were called ‘the phantom menace’ in the literature [7]. We emphasize the importance of routinely testing for ertapenem sensitivity as well as imipenem or meropenem in order to avoid missing detection of OXA-48-like carbapenemases in clinical settings.

**Table 1.** The clinical characteristics of the clinical strains

Patient	Age (years), sex	Diagnosis	Ward	Antibiotics	Strain	Isolation date	Specimen	ST	OXA	Other resistance genes
Patient 1	60, F	MDS, AML-M2	Haematology	IMP, LZD, VRC, VCV, MXF	YML0508	21 May 2018	Rectal swab	17	181	*bla*_SHV-94,_ qnrS1. oqxB. oqxA aac(6')-Ib-cr, fosA
Patient 2	84, M	Fracture, PE, HTN, Af, CRF, ARF	ICU	TZP	WSD411	26 August 2018	Rectal swab	15	232	*bla*_TEM-1B,_ *bla*_SHV-28,_ *bla*_CTX-M-15,_ aph(3')-Ia aac(6')-Ib3 aph(6)-Id rmtF, aph(3'')-Ib rmtB, aadA16 oqxB, oqxA, aac(6')-Ib-cr, qnrB1, fosA, floR, ARR-2, sul2
					WSD2016	12 September 2018	Faeces	76	232	*bla*_SHV-59,_ *bla*_TEM-1B,_ oqxB, oqxA, qnrS1, fosA, fosA3, floR, catA2, ARR-3, sul2, tet(A), dfrA27, aph(3'')-Ib, aph(6)-Id, rmtB
					WSD2080	22 October 2018	Faeces	76	232	*bla*_SHV-59,_ *bla*_TEM-1B,_ oqxB, oqxA, qnrS1, fosA, fosA3, floR, catA2, ARR-3, sul2, tet(A), dfrA27, aph(3'')-Ib, aph(6)-Id, rmtB

MDS, myelodysplastic syndrome; AML-M2, acute myelogenous leukaemia; PE, pulmonary embolism; HTN, hypertension; Af, atrial fibrillation; CRF, chronic renal failure; ARF, acute respiratory failure. IMP, imipenem; LZD, linezolid; VRC, voriconazole; VCV, valacyclovir; MXF, moxifloxacin; TZP, piperacillin-tazobactam. All the isolates were identified by the VITEK2 system and matrix assisted laser desorption ionization-time of flight (MALDI-TOF) MS.

**Table 2.** The antimicrobial susceptibility results of the clinical strains and transconjugants (mg l^−1^)

Strain	MIC (mg l^−1^)											
	IMP	MEM	ETP	CTX	FEP	LEV	CIP	AMK	GEN	ATM	TGC	CST
YML0508	0.5	0.25	4	0.25	0.25	1	1	1	1	0.125	0.5	0.125
YML0508-C	0.5	0.25	1.5	0.38	0.25	4	1	1	0.5	0.125	0.25	0.06
WSD411	4	4	>64	≥32	≥256	≥32	≥512	≥256	≥512	≥256	2	0.06
WSD411-C	0.5	0.125	1	0.125	0.25	0.19	0.25	1	0.25	0.125	0.5	0.06
WSD2016	0.5	0.25	8	0.75	1	0.5	0.5	1	1	0.125	0.25	0.125
WSD2016-C	0.5	0.125	0.5	0.19	0.25	0.25	0.25	1	0.5	0.25	0.125	0.125
WSD2080	0.5	0.5	4	0.25	0.5	0.5	0.5	1	1	0.125	0.25	0.125
WSD2080-C	0.5	0.125	0.38	0.064	0.25	2	1	1	0.5	0.125	0.25	0.06
EC600	0.125	0.06	0.006	0.094	0.125	0.25	0.25	1	0.5	0.125	0.125	0.06

YML0508/WSD411/WSD2016/WSD2080-C: strain EC600 acquired plasmid from strain YML0508/WSD411/WSD2016/WSD2080 by conjugation. IMP: imipenem; MEM: meropenem; ETP: ertapenem; CTX: cefotaxime; FEP: cefepime; CPS: cefperazone/sulbactam; LEV: levofloxacin; CIP: ciprofloxacin; ATM: aztreonam; TGC: tigecycline; CST: colistin. The shaded area represents the antimicrobial susceptibility results of clinical strains. The MICs for IMP, MEM, ETP, TGC and CST were determined using the broth microdilution method following the guidelines from the Clinical and Laboratory Standard Institute (CLSI). MICs were interpreted according to CLSI breakpoints for *K. pneumoniae*. Susceptibility to other antimicrobial agents was tested via etest (AB bioMérieux) or broth microdilution.

Genomic DNA from each of the *K. pneumoniae* isolates was sequenced on the Illumina HiSeq2000 platform. The mean coverage and N50 of contigs of assembly of whole-genome sequencing data was >90× and >48 kb, respectively (Table S1, available in the online version of the paper). According to multi-locus sequence typing (MLST) using the PubMLST database, YML0508 was sequence type (ST)17, WSD411 was ST15, and WSD2016 and WSD2080 were ST76. WSD2016 and WSD2080 have identical core genome (cg)MLST profiles while the other isolate from patient 2, WSD411, differs by 1990 alleles (Fig. 1). Draft genomes were screened for antibiotic resistance genes using ResFinder on the CGE server. This confirmed the presence of *bla*_OXA-181_ in YML0508, and *bla*_OXA-232_ in WSD411, WSD2016 and WSD2080. Additional antibiotic resistance genes consistent with phenotypic data were detected in each of the draft genomes (Table 1).

**Fig. 1.**
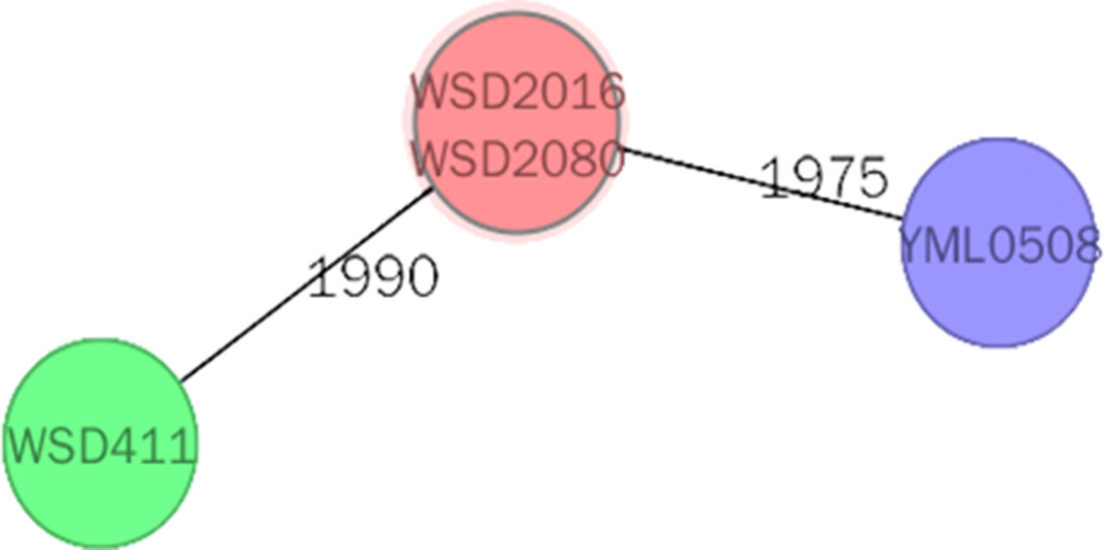
Minimum spanning tree based on cgMLST allelic profiles of four *K. pneumoniae* isolates. Each circle represents an allelic profile, and the numbers on the connecting lines illustrate the numbers of target genes with different alleles. The cgMLST scheme was executed as described in a previous study [17].

In the draft genomes of WSD411, WSD2016 and WSD2080, the *bla*_OXA-232_ gene was found in identical contigs with overlapping sequences at their ends. As such configurations represent circular plasmid sequences, one copy of the overlapping sequence was removed in each case. This revealed that the same 6141 bp plasmid, which was named pOXA-232_WSD, was present in all three isolates. According to PlasmidFinder, the replicon type of pOXA-232_WSD is ColKP3. pOXA-232_WSD does not contain any antibiotic resistance genes apart from *bla*_OXA-232_. The presence of an identical plasmid in two, unrelated *K. pneumoniae* STs within a single patient is clearly indicative of horizontal transfer. However, the transmission pathway could not be precisely determined with the data generated here.

The *bla*_OXA-181_ gene in the YML0508 draft genome was found in a short contig that did not represent a complete plasmid or chromosomal sequence. To determine the location of this gene, genomic DNA from YML0508 was sequenced using Oxford Nanopore MinION long-read sequencing technology. In the hybrid-assembled genome of YML0508, the *bla*_OXA-181_ gene was found in a 50 514 bp X3-type plasmid, designated pOXA-181_YML0508. pOXA-181_YML0508 also contained a partial ColKP3-type replicon, and the *qnrS1* gene associated with low-level resistance to fluoroquinolones. The sequence of pOXA-181_YML0508 was identical to many plasmids in the GenBank non-redundant nucleotide database, such as pOXA181_EC14828 (accession number KP400525), EC-p266917_2_04 (CP026727) and pOXA-181_29144 (KX523903). In pOXA-181_YML0508, the *bla*_OXA-181_ gene is part of a 3173 bp segment flanked on the left by a copy of IS*Kpn19* and on the right by a 2553 bp fragment of IS*3000* that has been truncated by a copy of IS*26* (Fig. 2). At either end of the segment that includes *bla*_OXA-181_ are fragments of the ColKP3 replication initiation gene, repA1Δ1(ColKP3) and repA1Δ2(ColKP3). The 3173 bp segment also includes a 264 bp fragment of IS*Ecp1*, and the entire region is identical to a region in plasmid pKP3-A (Fig. 2). Part of pOXA-232_WSD is also identical to this segment, apart from the one nucleotide substitution that distinguishes *bla*_OXA-181_ and *bla*_OXA-232_.

**Fig. 2.**
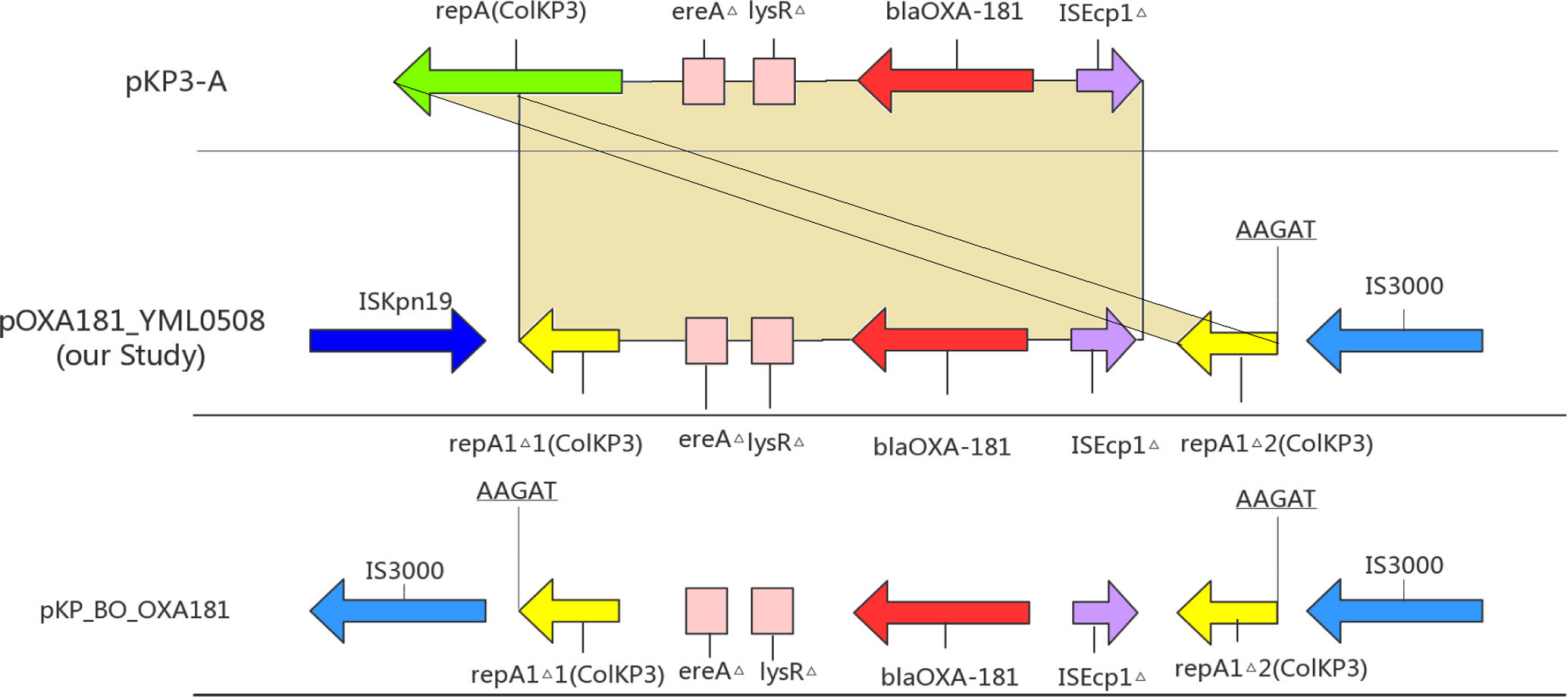
Scheme of the genetic environment of *bla*_OXA-181_ gene. △: incomplete. The yellow shadow represents the homology sequence between pKP3-A and pOXA-181.

This configuration surrounding *bla*_OXA-181_ in X3 plasmids has been described before [12]. In that study the role of IS*3000* in the co-integration of a ColKP3 plasmid into the X3 plasmid backbone was suspected, but could not be confirmed with the sequence data available at the time. However, using the sequence of this region from pOXA-181_YML0508 to query the GenBank nucleotide database revealed that a likely ancestral structure is found in *E. coli* plasmid pKP_BO_OXA181 (GenBank accession MG228426). In pKP_BO_OXA181, the ColKP3-derived sequence is flanked by two copies of IS*3000* (Fig. 2). In pKP_BO_OXA181, a 5 bp duplication (AAGAT) in the ColKP3-derived sequence shows that IS*3000* transposed into the ColKP3 backbone, indicating that IS*3000* is probably responsible for the integration of the ColKP3 plasmid into the X3 plasmid. The insertion sequence IS*Kpn19* in pOXA-181_YML0508 and other plasmids has probably inserted after the integration event, and subsequently deleted one copy of IS*3000*.

To evaluate the transferability and dissemination risk of the *bla*_OXA-181_- and *bla*_OXA-232_-containing plasmids from the clinical *K. pneumoniae* described here, conjugation experiments were performed using a filter mating method with rifampin-resistant *E. coli* EC600 as the recipient strain. Transconjugants were selected on Mueller-Hinton (MH) agar containing ampicillin (100 mg l^−1^) and rifampicin (1024 mg l^−1^). In all cases, the carbapenemase genes transferred from clinical *K. pneumoniae* to EC600. The mean conjugation frequencies of each plasmid, calculated from five independent experiments, were 8.13×10^−6^ transconjugants per donor for pOXA-232_WSD and 1.94×10^−4^ transconjugants per donor for pOXA-181_YML0508. The ertapenem MICs of transconjugants increased at least 60-fold relative to those of EC600, whereas the increase of the MICs of imipenem and meropenem were less pronounced, only about 2- to 8-fold (Table 2).

In this study, we screened four *K. pneumoniae* isolates harbouring *bla*_OXA-48_-like genes. One isolate, YML0508, contained the *bla*_OXA-181_ gene. The *bla*_OXA-181_ gene of YML0508 is in an X3 plasmid, and previous studies have described isolates of *K. pneumoniae *, *E. coli* and *K. variicola* [13, 14] that carry *bla*_OXA-181_-containing plasmids of the same type. We have provided strong evidence that the *bla*_OXA-181_ gene was acquired by an ancestral X3 plasmid along with a small ColKP3-type in an event facilitated by IS*3000*, and that IS*Kpn19* inserted later, before causing a deletion that complicated previous analyses of this region [12]. We also found an identical 6 141 bp ColKP3-type plasmid harbouring *bla*_OXA-232_ in ST15 and ST76 *K. pneumoniae* isolates from the same patient, suggesting horizontal transfer of this small plasmid. Consistent with this plasmid being transferrable, we showed that pOXA-232_WSD transferred from *K. pneumoniae* to *E. coli* in the laboratory at low frequency. As pOXA-232_WSD does not contain genes sufficient for self-transfer, it must utilize mobilization [15] for horizontal dissemination, and it will be interesting to determine which plasmid (or other element) might be mobilizing it. This is an important observation, as mobilization of relatively small plasmids is an underappreciated mechanism for carbapenemase gene dissemination. Furthermore, the presence of *bla*_OXA-232_, which produces a single amino acid variant of OXA-181, in a small plasmid might suggest that this mutation occurred in this context, as it is believed that the high copy number of small plasmids facilitates the emergence of antibiotic resistance gene variants [16]. The integration and transmission of plasmids with *bla*_OXA-48_-like genes contribute to the increasing prevalence of *K. pneumoniae* strains carrying *bla*_OXA-48_-like carbapenemases in China.

## Supplementary Data

Supplementary material 1
