# Supplementary material for: Emergence of carbapenem-resistant Klebsiella pneumoniae harbouring bla OXA-48-like genes in China
Source: J Med Microbiol. 2021 Jan 28;70(3):001306. doi: 10.1099/jmm.0.001306 (PMC8346730; doi:10.1099/jmm.0.001306)
Supplement: Supplementary material 1 [file jmm-70-306-s001.pdf]

Supplemental Table 1. The raw date presentation of whole-genome sequencing of clinical strains.

Strains	Coverage	N50 of contigs	GC content (%)	CDS no.	Accession no.
YML0508	270	377,036	57.30%	5,298	CP045193-CP045195
WSD411	90	48,371	55.20%	5,581	CP045686
WSD2016	129	183,232	55.10%	6,890	WIXS00000000
WSD2080	180	266,572	53.00%	6,123	WIXT00000000
